# Inhaled Asbestos Exacerbates Atherosclerosis in Apolipoprotein E–Deficient Mice via CD4^+^ T Cells

**DOI:** 10.1289/ehp.11172

**Published:** 2008-05-21

**Authors:** Naomi K. Fukagawa, Muyao Li, Tara Sabo-Attwood, Cynthia R. Timblin, Kelly J. Butnor, Jessica Gagne, Chad Steele, Douglas J. Taatjes, Sally Huber, Brooke T. Mossman

**Affiliations:** 1 Department of Medicine, University of Vermont College of Medicine, Burlington, Vermont, USA; 2 Department of Environmental Health Sciences, University of South Carolina, Columbia, South Carolina, USA; 3 Department of Pathology, University of Vermont College of Medicine, Burlington, Vermont, USA; 4 Department of Medicine, University of Alabama, Birmingham, Alabama, USA

**Keywords:** AP-1, atherosclerosis, CD4^+^ T-cells, chrysotile asbestos, fibrosis, inflammation, knockout mice, lung, MCP-1, NF-κB

## Abstract

**Background:**

Associations between air pollution and morbidity/mortality from cardiovascular disease are recognized in epidemiologic and clinical studies, but the mechanisms by which inhaled fibers or particles mediate the exacerbation of atherosclerosis are unclear.

**Objective and methods:**

To determine whether lung inflammation after inhalation of a well-characterized pathogenic particulate, chrysotile asbestos, is directly linked to exacerbation of atherosclerosis and the mechanisms involved, we exposed apolipoprotein E–deficient (ApoE^−/−^) mice and ApoE^−/−^ mice crossed with CD4^−/−^ mice to ambient air, NIEHS (National Institute of Environmental Health Sciences) reference sample of chrysotile asbestos, or fine titanium dioxide (TiO_2_), a nonpathogenic control particle, for 3, 9, or 30 days.

**Results:**

ApoE^−/−^ mice exposed to inhaled asbestos fibers had approximately 3-fold larger atherosclerotic lesions than did TiO_2_-exposed ApoE^−/−^ mice or asbestos-exposed ApoE^−/−^/CD4^−/−^ double-knockout (DKO) mice. Lung inflammation and the magnitude of lung fibrosis assessed histologically were similar in asbestos-exposed ApoE^−/−^ and DKO mice. Monocyte chemoattractant protein-1 (MCP-1) levels were increased in bronchoalveolar lavage fluid and plasma, and plasma concentrations correlated with lesion size (*p* < 0.04) in asbestos-exposed ApoE^−/−^ mice. At 9 days, activator protein-1 (AP-1) and nuclear factor-κB (NF-κB), transcription factors linked to inflammation and found in the promoter region of the *MCP-1* gene, were increased in aortas of asbestos-exposed ApoE^−/−^ but not DKO mice.

**Conclusion:**

Our findings show that the degree of lung inflammation and fibrosis does not correlate directly with cardiovascular effects of inhaled asbestos fibers and support a critical role of CD4^+^ T cells in linking fiber-induced pulmonary signaling to consequent activation of AP-1– and NF-κB–regulated genes in atherogenesis.

Cardiovascular disease is the leading cause of death and hospitalization among adults. An association between air pollution and morbidity/mortality from cardiovascular disease has been recognized in epidemiologic and clinical studies (Brook et al. 2004; Delfino et al. 2005; Dominici et al. 2006; Pope et al. 2002; Sun et al. 2005), but the mechanisms by which inhaled fibers or particles mediate the exacerbation of atherosclerosis are unclear. Moreover, elucidating the *in vivo* mechanisms of action of the multiple gaseous and particulate components of air pollution is challenging because of their physical and chemical complexity and dynamic nature of reactivity. Current theories purport that fine (≤ 2.5 μm in diameter) or ultrafine (≤ 0.1 μm in diameter) particles or their metal components (e.g., iron, vanadium) preferentially induce cardiovascular disease via nonspecific inflammation in lung, direct translocation to heart tissue, and site-specific effects or altered autonomic cardiac responses (Nel 2005; Oberdörster and Utell 2002; Pope et al. 2002; Pope and Dockery 2006).

The mechanisms of lung inflammation and disease by asbestos have been widely studied (Haegens et al. 2007; Janssen et al. 1997; Mossman et al. 2000) and its association with cardiovascular disease reported in occupational cohorts (Dement et al. 1983; McDonald et al. 1993; Sanden et al. 1993; Sjogren 1997, 2001). However, to our knowledge, no studies examining the mechanisms of the exacerbation of atherosclerosis by inhaled asbestos have been performed. Therefore, to test the hypothesis that airborne fibers exacerbate atherosclerosis and to elucidate the mechanisms involved, we used chrysotile asbestos fibers in a murine model of inhalation. Chrysotile asbestos is ubiquitous in the northern hemisphere and is the asbestos type historically used worldwide in > 95% of asbestos-containing products (Mossman et al. 1990). Although significant efforts have been made to reduce occupational and environmental exposure to amphibole types of asbestos (crocidolite, amosite) that may be more pathogenic in mesothelioma (Mossman and Gee 1989), the use of chrysotile asbestos continues worldwide (Nicholson 1997). Moreover, analysis of airborne surface dust in residential areas of Lower Manhattan after the collapse of the World Trade Center in New York City revealed increased chrysotile asbestos [Centers for Disease Control and Prevention (CDC) 2003], raising the concern about its short- and long-term pathogenic effects after inhalation by the general population.

In these experiments, we used the atherosclerosis-prone apolipoprotein E–deficient (ApoE^−/−^) mouse and ApoE^−/−^ mice crossed with CD4^−/−^ [double-knockout (DKO)] mice to test the hypothesis that inhaled asbestos fibers exacerbate atherosclerosis and that the mechanism involves CD4^+^ T cells that are increased in lung after inhalation of asbestos (Shukla et al. 2007). We exposed the mice to ambient air, fine titanium dioxide (TiO_2_; a nonpathogenic control particle), or chrysotile asbestos in the University of Vermont (UVM) Inhalation Facility (Sabo-Attwood et al. 2005). Our data are the first to show a direct relationship between inhaled pathogenic fibers and the exacerbation of atherosclerosis via a CD4^+^ T cell–dependent process. Moreover, they suggest an important role for activation of monocyte chemoattractant protein-1 (MCP-1) and early transcription factors activator protein-1 (AP-1) and nuclear factor-κB (NF-κB) in the development of atherosclerosis by inhaled pathogenic pollutants.

## Materials and Methods

### Animals

We maintained male and female breeding C57BL/6, ApoE^−/−^, and CD4^−/−^mice, purchased from Jackson Laboratories (Bar Harbor, ME), by brother/sister matings at UVM. We used the B6.129S2-Cd4^tm1Mak^ strain of CD4^−/−^ mice, the same background as the ApoE^−/−^ mice used in these experiments. Female offspring were used in our experiments. Double knockout (DKO) animals (ApoE^−/−^ and CD4^−/−^) were generated by producing F_2_ generation mice between ApoE^−/−^ and CD4^−/−^ and using polymerase chain reaction (PCR) to select mice that were homozygous for both gene knockouts. We then maintained homozygous ApoE^−/−^/CD4^−/−^DKO mice by brother/sister matings. Animals homozygous for both genes were used in the experiments. The DKO mice were selected in the F_2_ offspring by genotyping using PCR protocols and were backcrossed into the C57BL/6 background for more than eight generations. We maintained both strains as colonies at UVM and fed them normal chow. All procedures were approved by the UVM Institutional Committee on Use and Care of Animals. We treated mice humanely and with regard for the attenuation of suffering. We weighed all animals after anesthesia and found no differences between groups and treatments (data not shown).

### Inhalation procedures and sample collection

ApoE^−/−^ or DKO mice at 4–6 weeks of age were exposed to chrysotile asbestos [Mg_3_Si_2_O_5_(OH)_4_; National Institute of Environmental Health Sciences (NIEHS) reference sample at approximately 5 mg/m^3^ air; range, 4.7–5.7 mg/m^3^ air] for 6 hr/day, 5 days/week. This concentration is equivalent historically to concentrations of chrysotile asbestos in unregulated workplaces and levels that caused lung diseases, and in air during the World Trade Center disaster (CDC 2003). The size dimensions (mean aerodynamic diameter, 0.34 μm) of aerosolized NIEHS chrysotile asbestos have been reported previously (BéruBé et al. 1996).

We conducted whole-body inhalation exposures within our inhalation facility (accredited by the Association for Assessment of Laboratory Animal Care) as described previously (Sabo-Attwood et al. 2005). We exposed control animals (sham groups) to clean, ambient air. We studied younger animals with early lesion development to optimize the likelihood of detecting differences in lesion size as a consequence of exposure. If we had used older animals with larger lesions, lengths of exposure to asbestos fibers may not have elicited easily detectable differences in lesion size.

Because the schedule of exposure was 5 days/week, simulating a workplace setting, 30 days of exposure is equivalent to 6 weeks. We used shorter exposures to determine whether differences in early responses to asbestos might reveal potential mechanisms responsible for triggering differential lesion sizes at the end of 30 days of exposure, a time point previously shown to be associated with lung fibrogenesis (Sabo-Attwood et al. 2005).

We used fine TiO_2_ (0.2–2.5 μm diameter; Fisher Scientific, Pittsburgh, PA) as a non-pathogenic particle control at surface area concentrations approximately equal to those of asbestos (~ 28 mg/m^3^ air) in some of the experiments using ApoE^−/−^ mice. After 3, 9, or 30 days of exposure, we collected bronchoalveolar lavage fluid (BALF) and blood, aorta, and lung tissues and prepared them for analyses. We performed surgical procedures after injecting the mice intraperitoneally with sodium pentobarbital. We collected blood via cardiac puncture into tubes with sodium EDTA as the anticoagulant, and saved the plasma for chemokine/cytokine assays. Tracheas of mice were cannulated with polyethylene tubing, and the lungs lavaged with sterile calcium- and magnesium-free phosphate-buffered saline (PBS) in a total volume of 1 mL.

Mouse lung, heart, and aorta were dissected and immersion fixed overnight in 3% paraformaldehyde/PBS at 4°C as previously described (Taatjes et al. 2000; Wadsworth et al. 2002). During fixation, we bisected the hearts with a cut parallel to both atria. The hearts and lungs were immersed in optimal cutting temperature compound (OCT; Tissue-Tek, Torrance, CA) in labeled embedding molds (hearts were oriented cut side up so that the first sections taken would reveal the sinus area), snap-frozen in liquid-nitrogen–cooled 2-methyl butane, and stored at −80°C until the time of cryostat sectioning, as previously described (Paigen et al. 1987).

We defined the area for sectioning by three prominent valve cusps at the juncture of the aortic sinus region to the end of the valve region, when the valves disappeared and the aorta became more rounded in appearance.The sections on the slides were air-dried for 30 min to ensure proper adhesion before being stored in a slide box at −80°C. *En face* preparations were not done in these experiments because the thoracic and abdominal aortas were used for nuclear and cytoplasmic protein extraction for analyses of transcription factors.

### Aortic lesion quantitation

We examined oil red O–stained sections (Pearse method) using an Olympus BX50 upright light microscope (Olympus America, Inc., Lake Success, NY) with an attached Optronics MagnaFire digital camera (Optical Analysis Corp., Nashua, NH). The sections were imaged with a 4 × objective lens, and we used MagnaFire software (version 2.0) to capture 1,280 × 1,024 pixel RGB digital images. We performed computer-assisted image analysis using MetaMorph software (Universal Imaging Corp., Downingtown, PA) essentially according to previously published protocols (Wadsworth et al. 2002). We opened cropped digital images and set the appropriate (precalibrated) objective calibration by choosing “Measure/Calibrate Distance/Apply.” This allowed area measurements to be expressed in calibrated square micrometer values; pixel values were displayed at the bottom of the screen. The images were then assigned threshold values for pixel measurements. Once this was done, the “Integrated Morphometry” feature measured the thresholded area and logged the area values onto a Microsoft Excel spreadsheet (Microsoft Corp., Redmond, WA). The logged values were then converted into an Excel chart for presentation. Animal comparisons were calculated and expressed as area values.

### Fiber deposition studies in lung and aorta

To compare the fiber burden in lung with that in aorta, we digested the left lobes and aortas of two control and two asbestos-exposed mice in hypochlorite, a process that does not affect fiber integrity, as previously described (BéruBé et al. 1996). We transferred the digest to Nuclepore filters and examined them by scanning electron microscopy and X-ray energy-dispersive spectroscopy at 5,000× magnification. We evaluated 10 random fields per filter.

### Cholesterol measurement

We measured total cholesterol in mouse plasma in the early experiments using a standard commercial cholesterol esterase enzymatic assay (Cayman Chemical Co., Ann Arbor, MI). No differences in cholesterol levels were found between ApoE^−/−^ and DKO mice or in the different treatment groups.

### Lung histopathology for inflammation and fibrosis

To determine whether the extent of lung inflammation and fibrosis in lungs correlated with the extent of atherogenesis, lungs were inflated after collection of BALF with a 1:1 mixture of OCT and PBS and fixed in 4% paraformaldehyde. We stained paraffin sections of lung (5 μm thickness) with hematoxylin and eosin (H&E) to quantify inflammation or Masson’s trichrome technique for detection of collagen (Sabo-Attwood et al. 2005). A board-certified pathologist (K.J.B.) evaluated sections using a blind coding system, as previously described (Craighead 1982; Haegens et al. 2007).

We scored inflammation on a scale from 1 to 4: 1, no inflammation; 2, mild inflammation that was rarely peribronchiolar and consisted primarily of lymphocytes; 3, moderate inflammation with peribronchiolar neutrophils, eosinophils, lymphocytes, and abundant macrophages; and 4, severe inflammation with peribronchiolar neutrophils, eosinophils, and lymphocytes that extend to involve adjacent alveolar septa and abundant bronchiolar and intraalveolar macrophages. We also scored fibrosis on a scale from 1 to 4: 1, no fibrosis; 2, focal fibrosis; 3, moderate fibrosis; 4, severe fibrosis.

### BALF differential cell counts

We enumerated total cells in BALF and centrifuged 2 × 10^4^ cells onto glass slides at 600 rpm. We stained cytospins using the Hema3 kit (Biochemical Sciences, Swedesboro, NJ), and performed differential cell counts on 500 cells/mouse (Haegens et al. 2007).

### Cytokine concentrations in BALF and plasma

To determine whether altered cytokine profiles occurred in the lung and the systemic circulation, we initially measured cytokines using an enzyme-linked immunosorbent assay (ELISA). We used commercial kits for interleukin-6 (IL-6), MCP-1, macrophage inflammatory protein-2 (MIP-2), and interferon-γ (IFN-γ) (Endogen, Woburn, MA) according to the manufacturer’s directions. Briefly, we aliquoted 2-fold dilutions of plasma or BALF into 96-well microplates coated with antibody to the indicated cytokines for 2 hr at 25°C. Plates were then washed, incubated with biotin-conjugated secondary antibody, washed, incubated with streptavidin–horseradish peroxidase conjugate, washed again, and incubated with 3,3′,5,5′-tetramethylbenzidine substrate. The reaction was halted by adding the stop solution in the plate reader at 450 nm and analyzed using a Biotech EL808 ELISA Reader (BioTek, Inc., Winooski, VT). We determined cytokine concentrations from a standard curve using reference standards supplied in the kit.

In subsequent studies, we also measured cytokine and chemokine levels in BALF using the Bio-Plex Protein Array System and a mouse cytokine 22-plex panel (Bio-Rad, Hercules, CA), as previously described (Sabo-Attwood et al. 2005). This method of analysis is based on Luminex technology and simultaneously measures IL-1α, IL-1β, IL-2, IL-4, IL-5, IL-6, IL-9, IL-10, IL-12 (p40), IL-12 (p70), IL-13, IL-17, tumor necrosis factor-α (TNF-α), regulated in activation, normal T expressed and secreted (RANTES; CCL5), MIP-1α, MIP-1β, MCP-1, CXCR2 ligand KC/GRO-α (KC), granulocytecolony–stimulating factor (G-CSF), granulocyte/monocyte-colony–stimulating factor (GM-CSF), IFN-γ, and eotaxin protein. We determined concentrations of each cytokine and chemokine using Bio-Plex Manager software, Version 3.0 (Bio-Rad).

### Electrophoretic mobility shift assay (EMSA) and Western blot analyses for AP-1 and NF-κB in aortas

We used the EMSA to determine whether inhaled particulates affected the DNA binding of oxidant-associated transcription factors in aortic tissue. Isolated aorta was minced using sterile scissors and then homogenized in a prechilled homogenizer. Nuclear proteins were extracted as previously described (Janssen et al. 1997; Schreiber et al. 1989). Briefly, after homogenization, the cells were lysed in hypotonic buffer and 0.6% Nonidet P-40 (Sigma, St. Louis, MO). After centrifugation, the supernatant containing cytoplasmic proteins was collected and stored at −80°C for later analysis. Nuclear proteins were then extracted from the pelleted nuclei (Janssen et al. 1997; Schreiber et al. 1989).

Protein concentrations were measured using the Bio-rad Protein Assay (Bio-Rad). The AP-1 and NF-κB probes containing the consensus sequences of AP-1 and NF-κB binding sites, respectively, were synthesized commercially (Promega, Madison, WI). The oligos were then labeled with γ-^32^P-ATP (NEN Life Science, Boston, MA) by T_4_ polynucleotide kinase (GIBCO-BRL, Gaithersburg, MD). We incubated 20 μL of each binding mixture, composed of 7 μg nuclear protein in binding buffer with 40 mM HEPES (pH 7.8), 4% Ficoll 400, 200 μg/mL poly(dI:dC) [poly(deoxy-inosinic:deoxycytidylic acid); Amersham Pharmacia, Piscataway, NJ], 1 mM MgCl_2_, 0.1 mM dithiothreitol, and 1 μL of labeled probe (0.02 pmol), at room temperature for 15 min and then loaded it onto a 5% sodium dodecyl sulfate (SDS) polyacrylamide gel (nondenaturing), as previously described (Janssen et al. 1997; Mossman et al. 2000). The gel was run in 0.25 × Tris/borate/EDTA buffer at 120 V for 2.5 hr, then dried and exposed to X-ray film overnight at −80°C. The autoradiographic films were scanned by densitometry and analyzed with Quantity One Software, Version 4.2 (Bio-Rad).

We used cytoplasmic protein extracted from the aortas during the preparation of nuclear extracts for Western blot analysis to confirm the DNA binding of NF-κB by using an antibody to phosphorylated Kappa B inhibitor (IκB; Cell Signaling Technology, Inc., Danvers, MA). Briefly, 20 μg protein from each sample was electrophoresed on a 10% SDS-PAGE and electroblotted onto a nitrocellulose membrane, which was then incubated with the antibody overnight with shaking at 4°C. After incubation, the protein bands were visualized using a SuperSignal West Pico Trial Kit (Pierce, Rockford, IL) and exposed to radiographic film. The blots were reprobed with a β-actin antibody (Abcam, Cambridge, MA) to detect cytoplasmic β-actin as the loading control. The images and densities were captured with a GS-700 Imaging Densitometer (Bio-Rad), analyzed with Quantity One Software, and presented as the ratio of IκB to β-actin.

### Statistics

We used Student’s two-sample *t*-tests to compare groups when the data were normally distributed. We used the Mann-Whitney test to compare groups using transformed data to stabilize the variance or when results were not normally distributed. Two-way analysis of variance (ANOVA) was used to analyze transformed cytokine/chemokine data for each time point, with post hoc analysis to determine genotype effects within treatment or treatment effects within genotype. We used chi-square analysis to compare the histologic scores for fibrosis and inflammation. Nonparametric Kendall tau correlation coefficients were derived for the relationships between lesion size and MCP-1 concentrations in ApoE^−/−^ mice. All analyses were performed using SPSS, version 14, and data are presented as mean ± SE.

## Results

### Aortic responses

**Figure 1:**
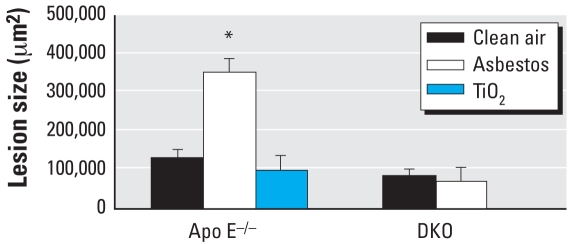
Size (mean ± SE) of atherosclerotic lesions after 30-day exposures of ApoE^−/−^ mice to clean air (*n* = 11), asbestos (*n* = 11), or TiO_2_ (*n* = 6), and of DKO mice to clean air (*n* = 8) or asbestos (*n* = 8). Error bars represent the variance in the average lesion area per animal. *p < 0.001.

We examined the effect of inhaled chrysotile asbestos fibers on the development of atherosclerotic lesions in three separate but identical inhalation experiments (*n* = 6–10 mice per group per time point per experiment). We observed no detectable differences in lesion size after 3- or 9-day exposures to asbestos. However, as determined by lipid staining with oil red O, lesions in the region of the aortic sinus to the aortic valve in 30-day asbestos-exposed ApoE^−/−^ mice, were approximately three times larger (*p* < 0.001 by ANOVA) than lesions in control, TiO_2_-exposed ApoE^−/−^ mice or in asbestos-exposed DKO animals (Figure 1). Fiber analyses showed only one or two small fibers in two fields of an aorta preparation from a clean-air control animal, which we concluded was likely caused by contamination from the previous processing of a lung sample. The rest of the aorta samples were free of fibers. These data suggest that asbestos-induced aortic effects are not due to direct translocation of fibers to aortic tissue.

### Lung responses

**Figure 2:**
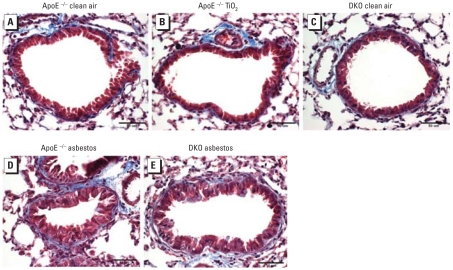
Representative lung sections stained with Masson’s trichrome after 30-day exposures, showing inflammatory cell infiltration and fibrosis in asbestos-exposed animals. Blue indicates collagen associated with fibrosis. (*A*) ApoE^−/−^ mice exposed to clean air. (*B*) ApoE^−/−^ mice exposed to TiO_2_. (*C*) DKO mice exposed to clean air. (*D*) ApoE^−/−^ mice exposed to asbestos. (*E*) DKO mice exposed to asbestos.

To determine whether the degree of lung inflammation and fibrosis in response to particulates correlated with the amount of atherosclerosis observed in the different groups of mice, we examined lung sections and graded them using an established blind coding system developed for asbestosis in human lung (Craighead 1982; Haegens et al. 2007). Figure 2 shows representative lung sections from a clean-air–exposed ApoE^−/−^ mouse (Figure 2A), a TiO_2_-exposed ApoE^−/−^ mouse (Figure 2B), and a clean-air–exposed DKO mouse (Figure 2C) after 30 days and demonstrates the absence of inflammation and fibrosis. In contrast, histologic analysis of lungs from both the ApoE^−/−^ (Figure 2D) and DKO (Figure 2E) mice showed striking peribronchiolar inflammation and fibrosis originating at distal bronchioles and alveolar duct regions after 30 days of asbestos exposure. As shown in Table 1 (which summarizes the grading of inflammatory cell infiltration) and Table 2 (which summarizes peribronchiolar/perivascular fibrosis) in H&E-stained lung sections, both ApoE^−/−^ and DKO mice had significantly greater immune cell infiltration after 3, 9, and 30 days of exposure to asbestos compared with exposure to clean air (*p* < 0.001). Fibrosis was not elevated until 30 days, and peribronchiolar fibrosis was significantly greater in asbestos-exposed ApoE^−/−^ and DKO mice compared with the respective clean-air controls (*p* < 0.001). We detected no differences in the fibrotic or inflammatory scores for lungs between asbestos-exposed ApoE^−/−^ and DKO mice at any time point.

**Figure 3:**
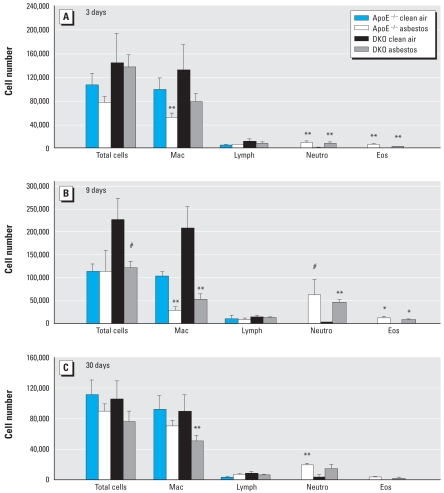
Differential cell counts (mean ± SE) in BALF from ApoE^−/−^ and DKO mice after exposure for 3 days (*A*), 9 days (*B*), or 30 days (*C*) to clean air or asbestos. Error bars represent the variance of the averages of the respective cell counts per animal. Eos, eosinophils; Lymph, lymphocytes; Mac, macrophages; Neutro, neutrophils. *p < 0.05, **p < 0.01, and #*p* < 0.001, compared with respective clean-air–exposed animals (*n* = 5–10 animals per group per treatment).

Figure 3 shows differential cell counts in BALF and also demonstrates similar changes in the inflammatory responses of ApoE^−/−^ and DKO mice after 3, 9, and 30 days of asbestos exposure compared with respective clean-air control animals. Total cell numbers and numbers of macrophages were higher in 9-day clean-air–exposed DKO mice, but the overall response to asbestos was similar to that in the 9-day ApoE^− /−^ mice. Asbestos exposure significantly increased total neutrophils and eosinophils in both ApoE^−/−^ and DKO mice at all time points.

### Cytokine and chemokine concentrations in BALF and plasma by ELISA

**Figure 4:**
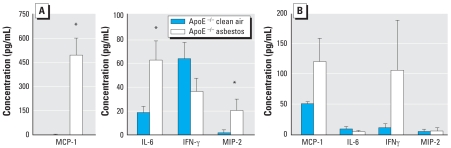
Cytokine concentrations (mean ± SE) in BALF (*A*) and plasma (*B*) obtained from ApoE^−/−^ mice exposed to clean air or asbestos for 30 days (*n* =11 per group). Error bars represent the variance of the averages of values obtained in each animal. *p < 0.04 compared with respective clean-air–exposed animals.

In initial experiments to determine whether cytokine and chemokine profiles in BALF or plasma correlated with atherogenic responses, we measured concentrations of four inflammatory cytokines (IL-6, IFN-γ, MCP-1, and MIP-2) previously associated with lung inflammation after asbestos exposure (Haegens et al. 2007; Sabo-Attwood et al. 2005), as well as the appearance of atherosclerosis (Hansson 2001) in BALF and in plasma obtained from ApoE^−/−^ mice exposed to clean air or chrysotile asbestos fibers for 30 days. In BALF, levels of MCP-1, IL-6, and MIP-2, a potent chemotactic factor for monocyte and neutrophil recruitment, were significantly increased (*p* < 0.04) in asbestos-exposed ApoE^−/−^ animals, whereas levels of IFN-γ were comparable in clean-air–exposed and asbestos-exposed groups (Figure 4A). We were also interested in whether plasma levels of these cytokines were elevated at 30 days, suggesting either systemic signaling to aortic tissue or their production by circulating inflammatory cells. In contrast to the other cytokines measured, only MCP-1 concentrations in plasma were significantly higher (*p* < 0.04) in asbestos-exposed compared with clean-air–exposed ApoE^−/−^ mice at 30 days (Figure 4B). Furthermore, plasma levels of MCP-1 significantly correlated with athero-sclerotic lesion sizes in asbestos-exposed ApoE^−/−^ mice (*p* < 0.04).

### Cytokine and chemokine levels in BALF and plasma using the Bio-Plex assay

**Figure 5:**
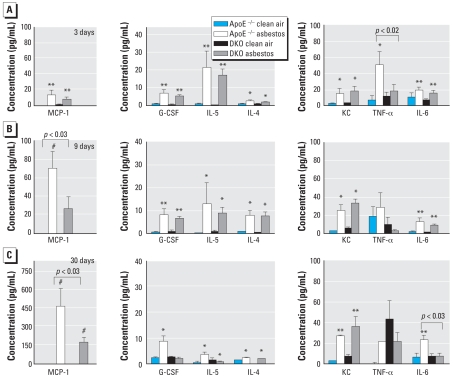
Cytokine and chemokine concentrations (mean ± SE) in BALF from ApoE^−/−^ and DKO mice exposed to clean air or chrysotile asbestos for 3 days (*A*), 9 days (*B*), or 30 days (*C*), as analyzed by the Bio-Plex assay. Error bars represent the variance of the averages of respective values from each animal. *p < 0.05, **p < 0.01, and #*p* < 0.001, compared with respective clean-air–exposed animals.

In later, more comprehensive experiments, we used the Bio-Plex assay for analysis of cytokines/ chemokines in BALF of asbestos-exposed ApoE^− / −^ and DKO mice after 3, 9, and 30 days of exposure. At 3 days, both ApoE^−/−^and DKO mice exposed to asbestos showed increased levels of MCP-1 (*p* < 0.01), G-CSF (*p* < 0.001), IL-5 (*p* < 0.01), IL-4 (*p* < 0.05), KC (*p* < 0.05), and IL-6 (*p* < 0.001) in BALF (Figure 5A). TNF-α in BALF was increased significantly only in ApoE^−/−^ mice exposed to asbestos (*p* < 0.05); levels were significantly higher in ApoE^−/−^ mice than in DKO mice (*p* < 0.02). The magnitudes of MCP-1 and IL-6 concentrations in BALF were similar in both ApoE^−/−^ and DKO mice after 3 days of asbestos exposure. However, none of the other cytokines on the panel were significantly altered at this time point.

After 9 days of asbestos exposure, which has previously been shown to be the time point of the maximum inflammatory response in lung after inhalation of chrysotile asbestos fibers (Haegens et al. 2007; Sabo-Attwood et al. 2005), MCP-1 in BALF remained elevated, with ApoE^−/−^ mice having an approximately 2-fold greater response than the DKO mice (*p* < 0.03) (Figure 5B). We observed increased BALF levels of G-CSF (*p* < 0.01), IL-5 (*p* < 0.05), IL-4 (*p* < 0.05), KC (*p* < 0.05), and IL-6 (*p* < 0.01) in both ApoE^−/−^ and DKO mice exposed to asbestos. Interestingly, the DKO mice had relatively lower TNF-α levels in BALF compared with the ApoE^−/−^ animals after either 3 or 9 days of exposure.

As shown in Figure 5C, significant elevations in BALF cytokines in both asbestos-exposed ApoE^−/−^ and DKO animals were sustained at 30 days for MCP-1 (*p* < 0.001), IL-4 (*p* < 0.05), and KC (*p* < 0.01). In contrast, only G-CSF (*p* < 0.05) and IL-6 (*p* < 0.01) levels remained significantly elevated in asbestos-exposed ApoE^−/−^ mice compared with clean-air–exposed animals. After asbestos exposure for 30 days, differences between ApoE^−/−^and DKO animals were found in BALF for MCP-1 (*p* < 0.03; mean ± SE, 491 ± 417 pg/mL in ApoE^−/−^ mice vs. 176 ± 107 pg/mL for DKO mice) and IL-6 (*p* < 0.03; 45 ± 46 pg/mL in ApoE^−/−^ vs. 7.4 ± 7.6 pg/mL in DKO mice) (Figure 5C).

Using the Bio-Plex assay on a subset of available ApoE^−/−^ and DKO plasma samples obtained in one 30-day exposure experiment, we found an approximately 49% increase in MCP-1 levels in the ApoE^−/−^ animals (clean air, 86 ± 30 pg/mL, *n* = 4; asbestos, 128 ± 51, *n* = 5). The magnitude of the change was similar to that found when using the ELISA assay. In contrast, we found no difference in MCP-1 concentrations between the clean-air–exposed and asbestos-exposed DKO mice (180 ± 9 pg/mL, *n* = 3, vs. 177 ± 70 pg/mL, *n* = 6, respectively). The levels in DKO mice were higher than those measured in ApoE^−/−^mice using both assays, but asbestos exposure did not elicit a change in the concentrations in plasma as it did in the ApoE^−/−^ mice. The Bio-Plex data followed patterns similar to those shown in Figure 4B for the ApoE^−/−^ mice. We found no differences in the clean-air–exposed versus asbestos-exposed DKO mice for IL-6 (0.7 ± 0.4 vs. 0.7 ± 1.5 pg/mL, respectively), TNF-α (29 ± 32 vs. 42 ± 54 pg/mL), or IFN-γ (0.2 ± 0.2 vs. 0.4 ± 0.6 pg/mL).

### AP-1 and NF-κB activation in the aorta

**Figure 6:**
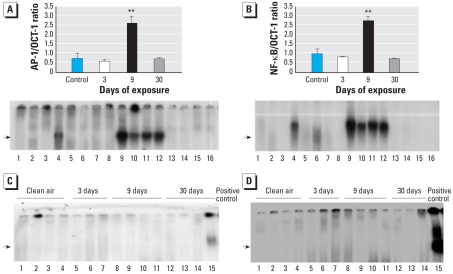
Results of EMSA for NF-κB and AP-1 in aortic extracts. (*A,B*) DNA binding of AP-1 (*A*) and NF-κB (*B*) in aortic extracts from ApoE^−/−^ mice exposed to clean air (control; lanes 1–4) or to chrysotile asbestos for 3 days (lanes 5–8), 9 days (lanes 9–12 ), or 30 days (lanes 13–16). Data are expressed relative to OCT-1 compound as a loading control. Values in top portions of *A* and *B* are mean ± SE; error bars represent the variance of the averages of values obtained for each animal. (*C,D*) DNA binding of AP-1 (*C*) and NF-κB (*D*) in aortic extracts from DKO mice exposed to clean air (lanes 1–4) or to asbestos for 3 days (lanes 5–7), 9 days (lanes 8–11), or 30 days (lanes 12–14). Lane 15 represents a positive control. Arrows denote bands for respective transcription factor binding. **p < 0.01 compared with control (*n* = 4 per group).

**Figure 7:**
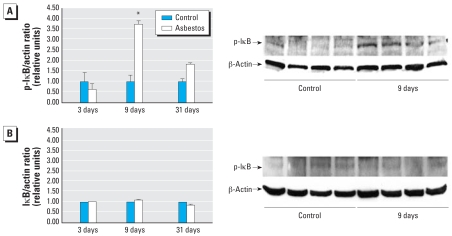
Results of Western blot analyses for phosphorylated IκB (p-IκB). (*A*) Western blot analyses (mean ± SE; *n* = 4 per group) for p-IκB/β-actin ratios in cytoplasmic extracts from aortas of ApoE^−/−^ mice exposed to clean air (control) or chrysotile asbestos for 3, 9, or 30 days (left), and an example of a Western blot comparing clean air with 9-day exposures (right). (*B*) Western blot analyses (mean ± SE; *n* = 4 per group) for p-IκB/β-actin ratios in cytoplasmic extracts from aortas of DKO mice exposed to clean air (control) or chrysotile asbestos for 3, 9, or 30 days (left), and an example of the Western blot comparing clean air with 9-day exposures (right). Blots are not shown for 3- and 30-day exposures because there were no significant changes. *p < 0.02 compared with clean air exposure.

Binding sites for both AP-1 and NF-κB (oxidant-induced transcription factors associated with inflammation) are found in the promoter region of the *MCP-1* gene, and their cooperative action is important in the induction of *MCP-1* gene expression (Martin et al. 1997). Although it is well known that both AP-1 and NF-κB are activated in the lung after asbestos exposure (Haegens et al. 2007; Janssen et al. 1997; Mossman et al. 2000) and that MCP-1 is important in the initiation of atherosclerosis (Gosling et al. 1999), distal changes in AP-1 and NF-κB binding to DNA in the aorta after inhalation of asbestos are novel findings. Data in Figure 6A and B demonstrate significant activation of both AP-1 and NF-κB, respectively, in the aorta from ApoE^−/−^ mice after 9 days of asbestos exposure, consistent both with patterns of early and significant inflammatory responses in lung tissue (Table 1) and with rises in MCP-1 levels in BALF (Figure 4). Figure 6C and D shows no AP-1 or NF-κB DNA binding, respectively, in aortas isolated from DKO mice exposed to clean air or asbestos. Western blot analyses for phosphorylated IκB in cytoplasmic protein extracted from the aortas also revealed increased levels relative to levels of β-actin, a loading control protein, after 9 days of asbestos exposure in ApoE^−/−^ mice (Figure 7A) but not in DKO mice (Figure 7B). Phosphorylated IκB releases the NF-κB complex for translocation to the nucleus; therefore, the increase in phosphorylated IκB found in the 9-day samples is consistent with the increased DNA binding activity of NF-κB in ApoE^−/−^ mice at this point as shown by EMSA (Figure 6).

## Discussion

These data are the first to show that inhaled chrysotile asbestos, a documented pathogenic airborne fiber, exacerbates atherosclerotic lesions in chow-fed ApoE^−/−^ mice. It should be noted that concentrations of airborne asbestos fibers generated in this study were high, as encountered episodically during the World Trade Center disaster, in contrast to mean ambient levels in buildings (0.00004 and 0.00243 fibers/mL air in rural and urban samples, respectively) or outdoor air (0.00001 and 0.0001 fibers/mL air in rural and urban samples, respectively) (Health Effects Institute 1991). Atherosclerotic effects were attenuated significantly when animals also lack CD4^+^ T cells, showing their critical importance in the pathogenesis of disease. The fact that DNA binding of both AP-1 and NF-κB were increased in nuclear extracts from aortas of ApoE^−/−^ mice after 9 days of asbestos exposure, in the absence of asbestos fibers in the aortas, indicates that distal signaling mechanisms involving these two transcription factors are activated in atherogenesis. We found no detectable differences in AP-1 and NF-κB DNA binding activity in extracts from aortas of DKO mice exposed to either clean air or asbestos, which provides additional support for the important interactions among asbestos exposure, CD4^+^ T cells, and lesion development through redox-sensitive signaling pathways. Furthermore, our studies suggest that MCP-1, which has binding sites for both AP-1 and NF-κB in its promoter region, is an important chemokine in the signaling events leading to atherosclerosis. Recently, Sun et al. (2005) reported that ApoE^−/−^ mice fed a high-fat diet and exposed to inhaled particulate matter had increased atherosclerotic lesions that were accompanied by increased macrophage infiltration and evidence of oxidative stress in the aorta. However, they did not identify the link between lung effects and these findings in aortic tissue and mechanisms of particulate matter–induced atherosclerosis.

Atherosclerosis is a complex disease with etiologies that involve both genetic and environmental factors. The present study confirms the importance of inflammation in the athero-genic process and supports a role for the CD4^+^ T cell, shown to be present in atherosclerotic lesions (Zhou et al. 1996). Although we did not stain for the presence of CD4^+^ T cells in lesions in these experiments, we did find more circulating CD4^+^ T cells in the blood of a small subset of ApoE^−/−^ mice exposed to asbestos compared with a subset exposed to clean air for 30 days, although the differences were not significant (data not shown). Early work using ApoE^−/−^ mice crossed with IFN-γ receptor–deficient mice suggested that IFN-γ promoted atherosclerosis and was critical in modulating the balance between T_H_1 (cellular immunity) and T_H_2 (humoral immunity) sub-sets of T cells (Gupta et al. 1997). Exogenous IFN-γ has also been shown to increase atherosclerosis in ApoE^−/−^ mice (Whitman et al. 2000). IL-4 is reportedly necessary for invoking a T_H_2 response (Feili-Hariri et al. 2005) and has been shown to play a role in the progression of atherosclerosis (Davenport and Tipping 2003). IL-4 levels were increased similarly in BALF of both ApoE^−/−^ and DKO mice exposed to asbestos for 3, 9, or 30 days, but levels were not detectable in plasma.

Elhage et al. (2004) reported that DKO mice had increased lesions in the descending and abdominal aorta, but they found similar lesions in the aortic sinuses of ApoE^−/−^ mice. We recognize that the lesions observed in the present study are larger than reported in the literature for ApoE^−/−^ mice of similar age. The reasons for the differences between the present work and previous reports are unclear but may relate to the study design and housing conditions. We chose to start the exposures to asbestos at 4–6 weeks of age and to terminate the experiments after 30 days of exposure (5 days/week, for a total of 6 weeks) when animals were at most 12 weeks of age. Because we examined only the aortic sinus, extending the exposures to 18 weeks or 1 year or doing *en face* analysis may have yielded different results. However, our objective was to determine whether the length of exposure to asbestos necessary to develop lung inflammation and early fibrosis was sufficient to elicit differences in aortic responses between ApoE^−/−^ and DKO mice. Of major significance here is that both ApoE^−/−^ and DKO mice appeared to respond similarly to asbestos exposure with respect to lung inflammation, fibrosis, and selected cytokine concentrations in BALF but differed significantly in their responses in plasma concentrations of MCP-1 after 30 days of exposure to asbestos. ApoE^−/−^ mice exposed to asbestos had an increase in plasma MCP-1 levels compared with clean-air–exposed animals. In contrast, MCP-1 concentrations in DKO mice, although constitutively higher, remained unchanged after clean air and asbestos exposure. In BALF, genotype differences in response became apparent at 9 and 30 days of exposure to asbestos in that MCP-1 and IL-6 were higher in ApoE^−/−^ than in DKO mice. The source of these cytokines remains unknown, and future studies will examine changes induced by asbestos in MCP-1–defi-cient mice crossed with ApoE^−/−^ mice.

Our data showing elevated levels of plasma MCP-1 in asbestos-exposed ApoE^−/−^ mice are consistent with growing evidence that cytokines participate as autocrine and paracrine mediators in the pathogenesis of atherosclerosis (Hansson 2001; Upadhya et al. 2004). Because elevated levels of MCP-1 are known to occur in BALF after inhalation of chrysotile asbestos in C57BL/6 mice (Haegens et al. 2007), neutrophils elaborating MCP-1 may enter the systemic circulation. Alternatively, cells activated in the lung by asbestos [e.g., macrophages, dendritic cells (DCs), neutrophils, lymphocytes] may migrate to pulmonary lymph nodes, where they activate CD4^+^ T cells that are released into the circulation and contribute to atherogenesis. This latter explanation could account for the remarkably similar inflammatory and fibrotic patterns in the lungs of ApoE^−/−^ and DKO mice, but the dramatically different extent of atherosclerotic lesions in their aortas. The elevation of systemic levels of MCP-1 and the significant relationship between plasma MCP-1 concentrations and lesion size in the ApoE^−/−^ mice provide support for previous work showing the importance of MCP-1 in atherogenesis (Gosling et al. 1999; Hansson 2001). DKO mice had higher plasma MCP-1 levels than did ApoE^−/−^ mice, but asbestos had no effect compared with clean air in these mice. This suggests that constitutive levels may be higher in DKO mice but that higher levels are not necessarily associated with larger lesions. It appears that the induced change in concentrations is of greater importance in the pathogenesis of the disease, highlighting the complex interrelationships.

Our work also demonstrates that many classically defined inflammatory cytokines/ chemokines (e.g., KC, MIP-1α, MIP-1β^,^ IL-6) are elevated in BALF in this model, but these differences appear to be dissociated from effects seen in the aorta. Previous studies have revealed that MCP-1 is necessary for the activation of T lymphocytes (Taub et al. 1995) and that MCP-1 produced by lung fibroblasts exerts an immunomodulatory effect on CD4^+^ T cells *in vitro* (Hogaboam et al. 1998). G-CSF and KC, both important in neutrophil recruitment, were elevated in our studies, but not GM-CSF, which is an important growth factor for lung DC maturation and proliferation (Vermaelen and Pauwels 2005). However, because DCs are central to the initiation and orchestration of immunity and tolerance, we suggest that asbestos fibers initiate an inflammatory cascade in lung epithelial cells and macrophages, cells first encountering inhaled asbestos, thus leading to secretion of cytokines and chemokines with DC-attracting potential. DCs then could migrate to lymph nodes, where they activate T cells, which enter the systemic circulation and come into contact with a vulnerable aorta, an observation consistent with the detection of macrophages and CD4^+^ T cells in early aortic lesions (Zhou et al. 1996). Further inflammatory cell infiltration would then exacerbate lesion development. Investigation is ongoing to document a role for DCs in the translation of asbestos-induced pulmonary inflammation to systemic responses.

In summary, exposure to inhaled chrysotile asbestos fibers was associated with an exacerbation of the development of atherosclerotic lesions in ApoE^−/−^ mice. The induction of atherosclerosis appears to be CD4^+^ T cell dependent and dissociated from the magnitude of lung inflammation or fibrosis associated with inhaled asbestos. More important, our data also suggest the importance of a change in systemic MCP-1 in atherogenesis associated with altered signaling through the transcription factors AP-1 and NF-κB, and that the effects of inhaled asbestos extend beyond the lung. The data also raise the possibility of constitutive differences in MCP-1 levels between the different genotypes that warrant further investigation.

## Figures and Tables

**Table 1:** Immune cell infiltration in lung tissue after asbestos exposure.^a^

	ApoE^−/−^		DKO	
Exposure time (days)	Control	Asbestos	Control	Asbestos
3	1.0 ± 0	2.0 ± 0*	1.0 ± 0	2.1 ± 0.2*
9	1.0 ± 0	2.8 ± 0.3*	1.0 ± 0	2.5 ± 0.5*
30	1.0 ± 0	3.5 ± 0.2*	1.0 ± 0	3.2 ± 0.2*

a Evaluated on H&E-stained sections using a scoring system where 1 indicates no infiltration; 2, mild infiltration; 3, moderate infiltration; and 4, severe infiltration.

* *p* < 0.001 compared with control.

**Table 2:** Fibrosis in lung tissue after asbestos exposure.^a^

	ApoE^−/−^			DKO		
		Asbestos			Asbestos	
Exposure time (days)	Control	Peribronchiolar	Perivascular	Control	Peribronchiolar	Perivascular
3	1.0 ± 0	1.0 ± 0	1.0 ± 0	1.0 ± 0	1.0 ± 0	1.0 ± 0
9	1.0 ± 0	1.5 ± 0.3	1.0 ± 0	1.0 ± 0	1.5 ± 0.5	1.5 ± 0.5
30	1.0 ± 0	1.8 ± 0.2*	1.3 ± 0.2	1.0 ± 0	2.3 ± 0.3*	1.0 ± 0

a Evaluated on lung sections stained with the Masson’s trichrome method to detect collagen, using a scoring system where 1 indicates no fibrosis; 2, focal fibrosis; 3, moderate fibrosis; and 4, severe fibrosis.

* *p* < 0.001 compared with control.

## References

[b1-ehp-116-1218] BéruBé KA, Quinlan TR, Moulton G, Hemenway D, O’Shaughnessy P, Vacek P (1996). Comparative proliferative and histopathologic changes in rat lungs after inhalation of chrysotile or crocidolite asbestos. Toxicol Appl Pharmacol.

[b2-ehp-116-1218] Brook RD, Franklin B, Cascio W, Hong Y, Howard G, Lipsett M (2004). Air pollution and cardiovascular disease: a statement for healthcare professionals from the Expert Panel on Population and Prevention Science of the American Heart Association. Circulation.

[b3-ehp-116-1218] CDC (Centers for Disease Control and Prevention) (2003). Potential exposures to airborne and settled surface dust in residential areas of lower Manhattan following the collapse of the World Trade Center—New York City, November 4-December 11, 2001. Morbid Mortal Wkly Rep.

[b4-ehp-116-1218] Craighead JE (1982). Asbestos-associated dieases: report of the Pneumoconiosis Committee of the College of American Pathologists and the National Institute for Occupational Safety and Health. Arch Pathol Lab Med.

[b5-ehp-116-1218] Davenport P, Tipping PG (2003). The role of interleukin-4 and interleukin-12 in the progression of atherosclerosis in apolipoprotein E-deficient mice. Am J Pathol.

[b6-ehp-116-1218] Delfino RJ, Sioutas C, Malik S (2005). Potential role of ultrafine particles in associations between airborne particle mass and cardiovascular health. Environ Health Perspect.

[b7-ehp-116-1218] Dement JM, Harris RL, Symons MJ, Shy CM (1983). Exposures and mortality among chrysotile asbestos workers. Part II: mortality. Am J Ind Med.

[b8-ehp-116-1218] Dominici F, Peng RD, Bell ML, Pham L, McDermott A, Zeger SL (2006). Fine particulate air pollution and hospital admission for cardiovascular and respiratory diseases. JAMA.

[b9-ehp-116-1218] Elhage R, Gourdy P, Brouchet L, Jawien J, Fouque MJ, Fiévet C (2004). Deleting TCRαβ^+^ or CD4^+^ T lymphocytes leads to opposite effects on site-specific atherosclerosis in female apolipoprotein E-deficient mice. Am J Pathol.

[b10-ehp-116-1218] Feili-Hariri M, Falkner DH, Morel PA (2005). Polarization of naive T cells into Th1 or Th2 by distinct cytokine-driven murine dendritic cell populations: implications for immunotherapy. J Leukoc Biol.

[b11-ehp-116-1218] Gosling J, Slaymaker S, Gu L, Tseng S, Zlot CH, Young SG (1999). MCP-1 deficiency reduces susceptibility to atherosclerosis in mice that overexpress human apolipoprotein B. J Clin Invest.

[b12-ehp-116-1218] Gupta S, Pablo AM, Jiang X, Wang N, Tall AR, Schindler C (1997). IFN-gamma potentiates atherosclerosis in ApoE knock-out mice. J Clin Invest.

[b13-ehp-116-1218] Haegens A, Barrett TF, Gell J, Shukla A, MacPherson M, Vacek P (2007). Airway epithelial NF-κB activation modulates asbestos-induced inflammation and mucin production in vivo. J Immunol.

[b14-ehp-116-1218] Hansson GK (2001). Immune mechanisms in atherosclerosis. Arterioscler Thromb Vasc Biol.

[b15-ehp-116-1218] Health Effects Institute (1991). Asbestos in Public and Commercial Buildings: A Literature Review and Synthesis of Current Knowledge. Special Report.

[b16-ehp-116-1218] Hogaboam CM, Lukacs NW, Chensue SW, Strieter RM, Kunkel SL (1998). Monocyte chemoattractant protein-1 synthesis by murine lung fibroblasts modulates CD4+ T cell activation. J Immunol.

[b17-ehp-116-1218] Janssen YM, Driscoll KE, Howard B, Quinlan TR, Treadwell M, Barchowsky A (1997). Asbestos causes translocation of p65 protein and increases NF-kappa B DNA binding activity in rat lung epithelial and pleural mesothelial cells. Am J Pathol.

[b18-ehp-116-1218] Martin T, Cardarelli PM, Parry GCN, Felts KA, Cobb RR (1997). Cytokine induction of monocyte chemoattractant protein-1 gene expression in human endothelial cells depends on the cooperative action of NF-κB and AP-1. Eur J Immunol.

[b19-ehp-116-1218] McDonald JC, Liddell FD, Dufresne A, McDonald AD (1993). The 1891–1920 birth cohort of Quebec chrysotile miners and millers: mortality 1976–88. Br J Ind Med.

[b20-ehp-116-1218] Mossman BT, Bignon J, Corn M, Seaton A, Gee JB (1990). Asbestos: scientific developments and implications for public policy. Science.

[b21-ehp-116-1218] Mossman BT, Gee JBL (1989). Asbestos-related diseases. N Engl J Med.

[b22-ehp-116-1218] Mossman BT, Hubbard A, Shukla A, Timblin CR (2000). Role of mitogen-activated protein kinases, early response protooncogenes, and activator protein-1 in cell signaling by asbestos. Inhal Toxicol.

[b23-ehp-116-1218] Nel A (2005). Atmosphere. Air pollution-related illness: effects of particles. Science.

[b24-ehp-116-1218] Nicholson WJ, Lehtinen S, Tossavainen A, Rantanen J (1997). Global analysis of occupational and environmental exposure to asbestos. People and Work.

[b25-ehp-116-1218] Oberdörster G, Utell MJ (2002). Ultrafine particles in the urban air: to the respiratory tract—and beyond? [Editorial]. Environ Health Perspect.

[b26-ehp-116-1218] Paigen B, Morrow A, Holmes PA, Mitchell D, Williams RA (1987). Quantitative assessment of atherosclerotic lesions in mice. Atherosclerosis.

[b27-ehp-116-1218] Pope CA, Burnett RT, Thun MJ, Calle EE, Krewski D, Ito K (2002). Lung cancer, cardiopulmonary mortality, and long-term exposure to fine particulate air pollution. JAMA.

[b28-ehp-116-1218] Pope CA, Dockery DW (2006). Health effects of fine particulate air pollution: lines that connect. J Air Waste Manage Assoc.

[b29-ehp-116-1218] Sabo-Attwood T, Ramos-Nino M, Bond J, Butnor KJ, Heintz N, Gruber AD (2005). Gene expression profiles reveal increased mClca3 (Gob5) expression and mucin production in a murine model of asbestos-induced fibrogenesis. Am J Pathol.

[b30-ehp-116-1218] Sanden A, Jarvholm B, Larsson S (1993). The importance of lung function, non-malignant diseases associated with asbestos, and symptoms as predictors of ischaemic heart disease in shipyard workers exposed to asbestos. Br J Ind Med.

[b31-ehp-116-1218] Schreiber E, Matthias P, Müller MM, Schaffner W (1989). Rapid detection of octamer binding proteins with ‘miniextracts’, prepared from a small number of cells. Nucleic Acids Res.

[b32-ehp-116-1218] Shukla A, Lounsbury KM, Barrett TF, Gell J, Rincon M, Butnor KJ (2007). Asbestos-induced peribronchiolar cell proliferation and cytokine production are attenuated in lungs of protein kinase C-δ knockout mice. Am J Pathol.

[b33-ehp-116-1218] Sjogren B (1997). Occupational exposure to dust: inflammation and ischaemic heart disease. Occup Environ Med.

[b34-ehp-116-1218] Sjogren B (2001). Association between pleural plaques and coronary heart disease [Letter]. Scand J Work Environ Health.

[b35-ehp-116-1218] Sun Q, Wang A, Jin X, Natanzon A, Duquaine D, Brook RD (2005). Long-term air pollution exposure and acceleration of atherosclerosis and vascular inflammation in an animal model. JAMA.

[b36-ehp-116-1218] Taatjes DJ, Wadsworth MP, Schneider DJ, Sobel BE (2000). Improved quantitative characterization of atherosclerotic plaque composition with immunohistochemistry, confocal fluorescence microscopy, and computer-assisted image analysis. Histochem Cell Biol.

[b37-ehp-116-1218] Taub DD, Proost P, Murphy WJ, Anver M, Longo DL, van Damme J (1995). Monocyte chemotactic protein-1 (MCP-1), -2, and -3 are chemotactic for human T lymphocytes. J Clin Invest.

[b38-ehp-116-1218] Upadhya S, Mooteri S, Peckham N, Pai RG (2004). Atherogenic effect of interleukin-2 and antiatherogenic effect of interleukin-2 antibody in apo-E-deficient mice. Angiology.

[b39-ehp-116-1218] Vermaelen K, Pauwels R (2005). Pulmonary dendritic cells. Am J Respir Crit Care Med.

[b40-ehp-116-1218] Wadsworth MP, Sobel BE, Schneider DJ, Taatjes DJ (2002). Delineation of the evolution of compositional changes in atheroma. Histochem Cell Biol.

[b41-ehp-116-1218] Whitman SC, Ravisankar P, Elam H, Daugherty A (2000). Exogenous interferon-gamma enhances atherosclerosis in apolipoprotein E−/− mice. Am J Pathol.

[b42-ehp-116-1218] Zhou X, Stemme S, Hansson GK (1996). Evidence for a local immune response in atherosclerosis. CD4+ T cells infiltrate lesions of apolipoprotein-E-deficient mice. Am J Pathol.

